# Family caregiver mistreatment of the elderly: prevalence of risk and associated factors

**DOI:** 10.1186/s12889-018-5067-8

**Published:** 2018-01-22

**Authors:** Francesc Orfila, Montserrat Coma-Solé, Marta Cabanas, Francisco Cegri-Lombardo, Anna Moleras-Serra, Enriqueta Pujol-Ribera

**Affiliations:** 1 Institut Universitari d’Investigació en Atenció Primaria Jordi Gol (IDIAP Jordi Gol), Gran Via de les Corts Catalanes, 587, 08007 Barcelona, Spain; 2 Gerència Territorial Barcelona Ciutat. Institut Catala de la Salut, Balmes 22, 08007 Barcelona, Spain; 3 Centre d’Atenció Primària Les Planes. Gerència Territorial Metropolitana Sud. Institut Catala de la Salut, 08970 Sant Joan Despí, Spain; 4 Consorci Sanitari de Barcelona, Esteve Terradas, 30, 08023 Barcelona, Spain; 5 Centre d’Atenció Primària Sant Martí. Gerència Territorial Barcelona Ciutat. Institut Catala de la Salut, Fluvià, 211, 08020 Barcelona, Spain; 6 Universitat Autònoma de Barcelona, 08193 Bellaterra (Cerdanyola del Vallès), Spain

**Keywords:** Elder abuse, Primary health care, Caregivers, Interpersonal relations, Risk factors

## Abstract

**Background:**

The detection of elder mistreatment is emerging as a public health priority; however, abusive behaviors exercised by caregivers are little known and rarely detected among primary health care professionals.

This study aims to estimate the prevalence of risk of abuse against community-residing elderly with moderate to severe dependency whose caregivers are relatives. In addition, we aim to describe the association between such a risk and socio-demographic variables, cognitive and dependency state of the victim, and the scale of the caregiver’s anxiety, depression, and burden.

**Methods:**

Cross-sectional study developed in 72 Primary Health Care teams from Barcelona, Spain. Participants were caregivers and their dependent care recipients (*N* = 829). Home interviews included the Caregiver Abuse Screen (CASE); self-reported abuse from care recipient; activities of daily living and cognitive state of the care recipient; anxiety and depression in caregivers and Caregiver Burden Scale. The relationship prior to the dependency, positive aspects of caregiving, and social support for the caregiver were also assessed. Multivariate analysis was performed using logistic regression with risk of abuse as dependent variable.

**Results:**

Caregivers were mainly women (82.8%) with a mean age of 63.3 years. Caregivers and care recipients lived in the same household in 87.4% of cases, and 86.6% had enjoyed a good previous relationship. Care recipients were women (65.6%), with a mean age of 84.2 years, and 64.2% had moderate to severe cognitive impairment. CASE demonstrated a prevalence of 33.4% (95% CI: 30.3-36.7) of abuse risk by the caregiver. Logistic regression showed as statistically significant: caregiver burden (OR = 2.75; 95% CI: 1.74-4.33), caregiver anxiety (OR = 2.06; 95% CI: 1.40-3.02), caregiver perception of aggressive behavior in the care recipient (OR = 7.24; 95% CI: 4.99-10.51), and a bad previous relationship (OR = 4.66; 95% CI: 1.25-17.4).

**Conclusions:**

Prevalence of risk of abuse is high among family caregivers. Our study has found risk factors in family caregivers that are preventable to an extent, namely: anxiety and feelings of burden. It is essential to become aware of these risk factors and their causes to intervene and help primary as well secondary prevention.

## Background

Elder abuse is influenced by multiple risk situations related to individual, relational, communal, and social factors. This type of mistreatment constitutes a violation of human rights and includes physical, sexual, psychological, emotional and financial abuse, and neglect, and therefore leads to a serious loss of dignity and respect [1–5]. It impacts negatively on the health of the victims, resulting in deterioration of the quality of life and existing medical conditions, depression and anxiety, increase in the number of hospital admissions and institutionalization, lower survival rates, and elevated social costs [6–9].

Elder abuse can take place in any setting, and can be inflicted by professionals or any citizen in general holding a position of trust. Nevertheless, it is most frequently perpetrated by those the care recipients confide in most, that is to say, their own caregivers. The detection and management of such mistreatment is emerging as a public health priority and a prime concern for health professionals despite its many challenges [2].

To develop effective preventive programs it is essential to increase the understanding of its causes, possible interventions [10], and ways to improve detection [11, 12]. Training and recognition can facilitate the identification and management by professionals and society of elder abuse [13] and raise victims’ awareness.

Prevalence of elder mistreatment varies in response to the definition of the problem, the methodology used, the measurement instruments, the setting, and the study population [10]. Two community studies in the United States showed a 10% prevalence of all types of abuse in individuals aged over 60 years [14, 15]. In contrast, in the same setting Burnes et al. [16] reported a figure of 4.6%, excluding financial abuse. According to the WHO Report for the European region, the prevalence of physical abuse against the elderly with disabilities, cognitive disorders and dependency reaches 25%, and family caregivers are involved in one third of the cases of mistreatment [17]. A review by Cooper et al. found a prevalence in the dependent elderly of 10.8% verbal abuse, 4.3% financial exploitation, 4.3% physical abuse, and 25% psychological abuse [18]. Data published by the National Center on Elder Abuse (NCEA) showed a prevalence of 47% in dementia patients [19] which concurred with another study published by Cooper et al. [20]. In Spain the prevalence of suspected mistreatment varies from 11.9% to 52.6% [21–25], nevertheless, Iborra et al. reported that only 4.6% of caregivers acknowledged having mistreated their care recipient at some point in time [26].

According to the social-ecological model, abuse is determined by individual aspects of the victim and the perpetrator, and also by relational, community, and social factors [12]. Negative effects of caring for a dependent relative such as stress, caregiver burden, mood disorders, and social isolation have been reported as risk factors for mistreatment of the care recipient. The personality of the caregiver, difficulty of care, and a challenging prior relationship are further aspects to be taken into account [26, 27]. Risk factors for the care recipient include: age over 74 years, female gender, intellectual or physical disability, dementia, and depression. Amongst the relational factors of the victim, social isolation and dependency constitute risk factors strongly associated with mistreatment according to most authors [1, 20, 28, 29].

A number of instruments have been developed to detect abuse. However, few have been validated [30] and many are too lengthy, or require too much training, to be implemented [31, 32]. Amongst the tools available and validated for use in caregivers is the Caregiver Abuse Screen (CASE) which evaluates possible physical/psychological mistreatment and neglect perpetrated by the caregiver. CASE is brief and well accepted by the interviewees because it does not generate the need for justification and therefore they do not adopt defensive attitudes [33]. The Spanish version developed by Gemma Pérez-Rojo et al. has shown sufficient validity and reliability [34].

This study aims to estimate the prevalence of mistreatment risk in a sample of the community-residing elderly with moderate to severe dependency whose caregivers are relatives. An additional objective is to describe the association between such a risk and socio-demographic variables, cognitive and dependency state of the victim, and the caregiver’s anxiety, depression, and burden.

## Methods

### Design and setting

This is a cross sectional study, part of a prospective cohort that analyzed the effect of the caregiving role on the health of family caregivers. It was conducted in a Primary Health Care setting. In Spain, Primary Health Care provides universal coverage and free access to health care for the entire population. We contacted the Primary Health Care Centers in the Barcelona province and we invited them to participate. The study includes a sample of family caregivers recruited between 2008 and 2010 from 72 Primary Health Care Centers.

### Participants

Participants were a convenience sample of 829 family caregivers that, for at least the past 3 years, had taken care in their own homes of individuals aged over 65 years of age with moderate to total dependency [35]. A structured interview was conducted in the home of the caregiver using validated instruments and complemented by a review of the medical records.

### Variables and measurement tools

Socio-demographic data of the caregiver and the care recipient were: age, gender, living in the same household, educational level (unfinished, primary, secondary, university studies), own income, and relationship (spouses, children, siblings, others).

#### Variables of the care recipient

Dependency level was measured with the Barthel index which evaluates 10 activities of daily living. Results are divided in 4 categories: total < 20, severe =20-35, moderate =40-55, mild = 60-95, and independent = 100 [35].

The Short Portable Mental Status Questionnaire (SPMSQ) assesses the subjects’ cognitive state [36]. Scores of 0-2 errors equate normal mental functioning; 3-4 errors indicate mild, 5-7 moderate, and 8-10 severe cognitive impairment.

#### Variables of the family caregivers

The Zarit Caregiver Burden Interview Short Form (7 items) screens for subjective perceptions of burden. The total score ranges from 5 to 45 [37]. A score ≥ 17 has been suggested as the cut-off point for high family-caregiving burden.

Assessment of mood with the Goldberg Anxiety and Depression Scale is based on nine depression and nine anxiety items [38]. Participants are considered likely to have anxiety with scores of 4 or more and depression with scores of 2 or more.

Personal resources: To assess the perception of positive aspects of caregiving (PAC), participants responded to the instrument of Tarlow et al. [39], a nine-item positive aspect of caregiving Likert-type scale. The total score ranges from 5 to 45, higher scores indicate more positive appraisals.

The study also measured the perception of the caregivers’ previous relationship with the care recipients (very good, good, fair, poor, and very poor). Instrumental social support was assessed by asking whether they could rely on somebody to attend to the needs of the care recipients if they were unable to do so.

Situations of mistreatment risk on the part of the caregiver were measured with the CASE screening tool validated in English by Reis and Namiash [33] and in Spanish by Pérez-Rojo [34]. It is made up of 8 questions with binary answers: risk of abuse is established by a positive score in four or more questions. CASE has been specifically designed to be used in the community and to evaluate physical and psychological abuse (items 1-4, 6 and 8) and neglect (items 5 and 7). The interview is conducted in a friendly environment where the answers provided by the caregiver are assessed and contextualized.

The caregivers’ perception of aggressive behavior in the care recipient was evaluated by means of a five-item questionnaire designed ad hoc by the authors of this study (Appendix 1).

### Statistical analysis

Logistic regression models were used to identify factors associated with risk of abuse and neglect by the caregiver according to CASE. Gender, age groups of the caregiver and the care recipient, previous relationship, PAC perception, social support, the SPMSQ, Barthel, Golberg and Zarit tools, and aggressive behavior of the care recipient toward the caregiver were included [40]. Data are presented as crude odds ratios, first with all variables forced into a model, and then a final model with significant variables selected using a forward conditional approach.

Confidence level was set at 95% and the level of statistical significance at 0.05. SPSS for Windows version 18 was used for the statistical analysis.

## Results

A total of 829 interviews with family caregivers were analyzed. The caregivers’ mean age was 63.3 years and 82.8% were women, their characteristics are described in Table 1. The average number of years spent caregiving was 8.4 and this activity occupied most of their days; 23.4% of the caregivers could not count on anybody in case of need. The Goldberg scale revealed that 59% of them were at risk of depression, 55% at risk of anxiety, and 68.2% presented feelings of burden according to Zarit’s Burden Interview.

**Table 1:** Caregiver characteristics and risk of abuse

		Total sample	Total Risk of abuse		Physical/psychological		Neglect	
		*N* = 829 *N* (%)	High *N* (%)	Low *N* (%)	High *N* (%)	Low *N* (%)	High *N* (%)	Low *N* (%)
Gender *N* = 829	Male	143 (17.2)	41 (28.7)	102 (71.3)	45 (31.5)	98 (68.5)	71 (49.7)	72 (50.3)
	Female	686 (82.8)	236 (34.4)	450 (65.6)	255 (37.2)	431 (62.8)	379 (55.2)	307 (44.8)
Age (years) *N* = 829	20-44 y.	44 (5.3)	18 (59.1)	26 (40.9)	19 (43.2)	25 (56.8)	21 (47.7)	23 (52.3)
	45-64 y.	433 (52.2)	145 (33.5)	288 (66.5)	163 (37.6)	270 (62.4)	239 (55.2)	194 (44.8)
	65-74 y.	179 (21.6)	66 (36.9)	113 (63.1)	68 (38.0)	111 (62.0)	91 (50.8)	88 (49.2)
	75+ y	173 (20.9)	48 (27.7)	125 (72.3)	50 (28.9)	123 (71.1)	99 (57.2)	74 (42.8)
Live together *N* = 812	Yes	710 (87.4)	240 (33.8)	470 (66.2)	258 (36.3)	452 (63.7)	386 (54.4)	324 (45.6)
	No	102 (12.6)	33 (32.4)	69 (67.6)	38 (37.3)	64 (62.7)	60 (58.8)	42 (41.2)
Relationship *N* = 829	Partner	265 (32.0)	86 (32.5)	179 (67.5)	90 (34.0)	175 (66.0)	153 (57.7)	112 (42.3)
	Children	481 (58.0)	162 (33.7)	319 (66.3)	176 (36.6)	305 (63.4)	253 (52.6)	228 (47.4)
	Siblings	21 (2.5)	8 (38.1)	13 (61.9)	8 (38.1)	13 (61.9)	11 (52.4)	10 (47.6)
	Others	62 (7.5)	21 (33.9)	41 (66.1)	26 (41.9)	36 (58.1)	33 (52.2)	29 (49.8)
Education *N* = 822	Incomplete	493 (60.0)	167 (33.9)	326 (66.1)	181 (36.7)	312 (63.3)	265 (53.8)	228 (46.2)
	Primary	116 (14.1)	37 (31.9)	79 (68.1)	44 (37.9)	72 (62.2)	61 (52.6)	55 (47.4)
	Secondary	159 (19.3)	55 (34.6)	104 (65.4)	58 (36.5)	101 (63.5)	92 (57.9)	67 (42.1)
	University	54 (6.6)	16 (29.6)	38 (70.4)	15 (27.8)	39 (72.2)	28 (51.9)	26 (48.1)
Remunerated work *N* = 818	Yes	179 (21.9)	67 (37.4)	112 (62.6)	71 (39.7)	108 (60.3)	104 (58.1)	75 (41.9)
	No	639 (78.1)	208 (32.6)	431 (67.4)	226 (35.4)	413 (64.6)	340 (53.2)	299 (46.8)
Own income *N* = 821	Yes	525 (63.9)	184 (35.0)	341 (65.0)	188 (35.8)	337 (64.2)	290 (55.2)	235 (44.8)
	No	296 (36.1)	90 (30.4)	206 (69.6)	109 (36.8)	187 (63.2)	155 (52.4)	141 (47.6)
Years of care *N* = 817	Mean (SD)	8.4 (7.4)	8.3 (6.3)	8.4 (7.8)	8.1 (6.3)	8.6 (7.9)	8.7 (7.6)	8.1 (7.0)
Hours a day dedicated to care *N* = 822	Mean (SD)	17.6 (7.5)	17.7 (7.1)	17.6 (7.7)	17.5 (7.2)	17.7 (7.7)	17.7 (7.5)	17.6 (7.7)
Previous relationship *N* = 805	Good	701 (87.1)	216 (30.8)**	485 (69.2)	230 (32.8)**	471 (67.2)	370 (52.8)*	331 (47.2)
	Fair	84 (10.4)	41 (48.8)	43 (51.2)	48 (57.1)	36 (42.9)	50 (59.5)	34 (40.5)
	Poor	20 (2.5)	17 (85.0)	3 (15.0)	18 (90.0)	2 (10.0)	16 (80.0)	4 (20.0)
Positive aspects of care *N* = 816	1st quartile (min)	188 (23.0)	91 (48.4)**	97 (51.6)	91 (48.4)**	97 (51.6)	127 (67.6)**	61 (32.4)
	2nd quartile	215 (26.3)	79 (36.7)	136 (63.3)	94 (43.7)	121 (56.3)	104 (48.4)	111 (51.6)
	3rd quartile	173 (21.2)	50 (28.9)	123 (71.1)	52 (30.1)	121 (69.9)	109 (63.0)	64 (37.0)
	4th quartile (max)	240 (29.4)	52 (21.7)	188 (78.3)	59 (24.6)	181 (75.4)	104 (43.3)	136 (56.7)
Has someone to count on *N* = 815	Yes	624 (76.6)	198 (31.7)*	426 (68.3)	215 (34.5)*	409 (65.5)	330 (52.9)*	294 (47.1)
	No	191 (23.4)	79 (41.4)	112 (58.6)	82 (42.9)	109 (57.1)	118 (61.8)	73 (38.2)
Zarit burden scale *N* = 820	Burden	559 (68.2)	239 (42,8)**	320 (58.6)	248 (44.4)	311 (56.6)	363 (64.9)**	196 (35.1)
	No burden	261 (31.8)	35 (13.4)	226 (86.6)	49 (18.8)	212 (81.2)	80 (30.7)	181 (69.3)
Goldberg depression scale *N* = 785	Depressed	463 (59.0)	190 (41.1)**	273 (59.9)	190 (41.0)**	273 (59.0)	300 (64.8)**	163 (35.2)
	Not depressed	322 (41.0)	69 (21.4)	253 (78.6)	90 (28.0)	232 (72.0)	123 (38.2)	199 (61.8)
Goldberg anxiety scale *N* = 813	Anxious	448 (55.1)	205 (45.8)**	243 (54.2)	210 (46.9)**	238 (53.1)	300 (67.0)**	148 (33.0)
	Not anxious	365 (44.9)	71 (19.5)	294 (80.5)	89 (24.4)	276 (75.6)	144 (39.5)	221 (60.5)
Caregivers’ perception of aggressive behavior in the care recipient *N* = 829	1+ positive responses	404 (48.7)	225 (55.7)**	179 (44.3)	246 (60.9)**	158 (39.1)	258 (63.9)**	146 (36.1)
	0 positive responses	425 (51.3)	52 (12.2)	373 (87.8)	54 (12.7)	371 (87.3)	192 (45.2)	233 (54.8)

**Positive aspects of caregiving scale** of 9 items (range 5 to 45). Higher scores indicate greater perceived PAC.; **Goldberg depression** of 9 items (range from 0 to 9). Values ≥2 have 50% chance of having clinically important disturbance. **Goldberg Anxiety** of 9 items (range from 0 to 9). Values ≥4 have probability of having clinically important disturbance. **Zarit Burden Scale** of 9 items. Values range from 5 to 45. Higher scores indicate more burden. *p < 0.05; **p < 0.001

The characteristics of the care recipients are described in Table 2. The average age was 84.2 years and 65.6% were women. Scores equivalent to moderate or severe cognitive impairment were found in 64.2%, and 67.5% presented severe or total dependency.

**Table 2:** Care recipient characteristics and risk of abuse

		Total sample	Total Risk of abuse		Physical/psychological		Neglect	
		*N* = 829 *N* (%)	High *N* (%)	Low N (%)	High *N* (%)	Low *N* (%)	High *N* (%)	Low *N* (%)
Gender *N* = 829	Male	285 (34.4)	92 (32.3)	193 (67.7)	99 (34.7)	186 (65.3)	164 (57.5)	121 (42.5)
	Female	544 (65.6)	185 (34.0)	359 (66.0)	201 (36.9)	343 (63.1)	286 (52.6)	258 (47.4)
Age (years) *N* = 829	< 80 y.	218 (26.3)	78 (35.8)	140 (64.2)	81 (37.2)	137 (62.8)	133 (61.0)	85 (39.0)
	80-89 y.	386 (46.6)	129 (33.4)	257 (66.6)	142 (36.8)	244 (63.2)	205 (53.1)	181 (46.9)
	90+ y.	225 (27.1)	70 (31.1)	155 (68.9)	77 (34.2)	148 (65.8)	112 (49.8)	113 (50.2)
Barthel Index *N* = 829	Moderate dependence	269 (32.4)	116 (43.1)**	153 (56.9)	123 (45.7)**	146 (54.3)	160 (59.5)	109 (40.5)
	Severe dependence	235 (28.3)	76 (32.3)	159 (67.7)	89 (37.9)	146 (62.1)	121 (51.5)	114 (48.5)
	Total dependence	325 (39.2)	85 (26.2)	240 (73.8)	88 (27.1)	237 (72.9)	169 (52.0)	156 (48.0)
SPMSQ *N* = 817	Intact cognition	159 (19.5)	55 (34.6)	104 (65.4)	54 (34.0)	105 (66.0)	94 (59.1)*	65 (40.9)
	Mild impairment	134 (16.4)	56 (41.8)	78 (58.2)	62 (46.3)	72 (53.7)	80 (59.7)	54 (40.3)
	Moderate impairment	199 (24.4)	72 (36.2)	127 (63.8)	78 (39.2)	121 (60.8)	113 (56.8)	86 (43.2)
	Severe impairment	325 (39.8)	94 (28.9)	231 (71.1)	105 (32.3)	220 (67.7)	156 (48.0)	169 (52.0)

**Barthel Index.** Values range from 0 to 100. Levels of dependence: Total < 20, Severe =20-35, Moderate =40-55. **SPMSQ: Short Portable Mental Status Questionnaire.** Values range from 0 to 10. Scores of 0-2 errors equate intact cognition; 3 to 4 errors indicate mild, 5 to 7 moderate and 8 to 10 severe cognitive impairment. *p < 0.05; **p < 0.001

Table 3 shows the caregivers’ responses to the CASE questionnaire. The prevalence of high risk of mistreatment (≥ 4 positive answers) was 33.4% (95% CI: 30.3-36.7), with an average of 2.7 positive answers. The physical and psychological risk of abuse component (6 items) obtained an average of 1.9 positive answers, with 36.2% of the caregivers providing 3 or more positive answers. The component of neglect (2 items) obtained an average of 0.76 positive answers, with 54.3% of the caregivers replying with one or two positive answers.

**Table 3:** Caregiver Abuse Screen (CASE)^a^ responses

	Total = 829 N (%)
Positive responses	
Item 1. Do you sometimes have trouble making _ control his/her temper or aggression?	376 (45.4)
Item 2. Do you often feel you are being forced to act out of character or do things you feel bad about?	270 (32.6)
Item 3. Do you find it difficult to manage _‘s behavior?	269 (32.4)
Item 4. Do you sometimes feel that you are forced to be rough with _?	350 (42.2)
Item 5. Do you sometimes feel you can’t do what is really necessary or what should be done for _?	300 (36.2)
Item 6. Do you often feel you have to reject or ignore _?	171 (20.6)
Item 7. Do you often feel so tired and exhausted that you cannot meet _‘s needs?	326 (39.3)
Item 8. Do you often feel you have to yell at _?	180 (21.7)
CASE > = 4 positive responses	277 (33.4)
CASE positive responses, mean (DE)	2.7 (2.3)
Physical/psychological abuse dimension > = 3 positive responses	300 (36.2)
Physical/psychological abuse dimension, mean (DE)	1.95 (1.9)
Neglect dimension > = 1 positive responses	443 (54.3)
Neglect dimension, mean (DE)	0.76 (0.8)

^a^Caregiver Abuse Screen (CASE). 8 items with binary answers (range from 0 to 8)

- Risk of abuse: positive score in four or more questions

- Risk of physical and psychological abuse: positive score in three or more questions (items 1-4, 6 and 8)

- Risk of neglect: positive score in one or more questions (items 5 and 7)

Table 1 also depicts the characteristics of the caregivers according to risk and type of mistreatment (physical/psychological and neglect). Caregivers with a higher perception of positive aspects of care, and those that had had a prior good relation with the care recipient, presented a lesser global risk of abuse and diminished risk for the physical/psychological and neglect components (*P* < 0.01).

Caregiving burden, anxiety, and depression were associated with a higher risk of abuse (*P* < 0.005) in addition to aggressive behavior on the part of the care recipient (*P* < 0.001). Such aggressive behavior was less frequent when the care recipient dependency was total than when it was moderate or severe (37.5% vs 56%; *P* < 0.001).

Total functional dependency was associated with a lower risk of mistreatment than moderate dependency in the global questionnaire as well as in the physical-psychological components. Cognitive impairment in the care recipient was related to a lower risk of neglect (Table 2).

In the final logistic regression model with the dependent variable being risk of abuse (yes/no), the following variables were statistically significant: aggressive behavior from the care recipient (OR = 7.24), a difficult previous relationship (OR = 4.66), caregiver’s perception of burden (OR = 2.75), and caregiver’s anxiety (OR = 2.06) (Table 4).

**Table 4:** Risk of abuse and associated factors. Logistic regression models: univariate, multivariate with all the variables and multivariate with significant variables

		Crude Univariate LR^a^	Multivariate LR Enter Method	Multivariate LR Final Model^d^
Variables		OR^b^ (IC95%)^c^	OR (IC95%)	OR (IC95%)
Gender, caregiver	Male	1	1	
	Female	1.31 (0.88-1.94)	0.97 (0.56-1.68)	
Age, caregiver	20-44 y.	1	1	
	45-64 y.	0.73 (0.39-1.37)	0.54 (0.23-1.26)	
	65-74 y.	0.84 (0.43-1.65)	0.78 (0.32-1.90)	
	75+ y	0.54 (0.27-1.08)	0.51 (0.20-1.27)	
Gender, care-recipient	Male	1	1	
	Female	1.08 (0.80- 1.47)	1.1 (0.70-1.71)	
Age, care-recipient	< 80 y.	1	1	
	80-89 y.	0.90 (0.64-1.28)	0.78 (0.48-1.29)	
	90+ y	0.81 (0.55-1.20)	0.57 (0.32-0.99)	
Previous relationship	Good/Very good	1	1	1
	Fair	2.14 (1.36-3.38)	1.50 (0.85-2.65)	1.54 (0.90-2.62)
	Poor/Very poor	12.72 (3.69-43.87)	4.13 (1.04-16.36)	4.66 (1.25-17.42)
Positive aspects of caregiving scale	1st quartile (min)	1	1	
	2nd quartile	0.62 (0.42-0.92)	0.61 (0.37-1.02)	
	3rd quartile	0.43 (0.28-0.67)	0.56 (0.32-0.98)	
	4th quartile (max)	0.30 (0.19-0.45)	0.58 (0.34-0.99)	
Has someone to count on	No	1	1	
	Yes	0.66 (0.47-0.92)	0.72 (0.47-1.12)	
Short Portable Mental Status Questionnaire	Intact cognition	1	1	
	Mild Impairment	1.36 (0.85-2.18)	1.25 (0.67-2.31)	
	Moderate Impairment	1.07 (0.69-1.66)	1.08 (0.61-1.91)	
	Severe Impairment	0.77 (0.51-1.15)	1.20 (0.69-2.07)	
Barthel Index	Moderate dependence	1	1	
	Severe dependence	0.63 (0.44-0.91)	0.63 (0.39-1.00)	
	Total dependence	0.47 (0.33-0.66)	0.58 (0.36-0.92)	
Goldberg depression	No	1	1	
	Yes	2.55 (1.85-3.53)	0.97 (0.62-1.53)	
Goldberg Anxiety	No	1	1	1
	Yes	3.49 (2.54-4.81)	1.91 (1.22-2.98)	2.06 (1.40-3.02)
Zarit Burden Scale	No burden	1	1	1
	Burden	4.82 (3.25-7.15)	2.80 (1.68-4.68)	2.75 (1.74-4.34)
Caregivers’ perception of aggressive behavior in the care recipient	No	1	1	1
	Yes	9.02 (6.35-12.80)	7.05 (4.70-10.58)	7.24 (4.99-10.52)

^a^*LR* Logistic regression, ^b^*OR* Odds Ratio, ^c^*IC95%* 95% confidence interval. ^d^Forward conditional approach Hosmer & Lemeshow test sig:0.74; *N* = 788

***Positive aspects of caregiving scale*** of 9 items (range 5 to 45). Higher scores indicate greater perceived PAC

***Short Portable Mental Status Questionnaire*** *of* 10 items (range 0 from 10). Higher scores indicate worse mental status

***Barthel Index****.* Values range from 0 (completely dependent) to 100 (completely Independent)

***Goldberg depression*** of 9 items (range from 0 to 9). Values ≥2 have 50% chance of having clinically important disturbance. ***Goldberg Anxiety*** of 9 items (range from 0 to 9). Values ≥5 have 50% chance of having clinically important disturbance

***Zarit Burden Scale*** of 9 items. Values range from 5 to 45. Higher scores indicate more burden

In the multivariate logistic regression models analyzing risk of mistreatment subtypes and associated factors, the significant variables related to physical/psychological abuse were: aggressive behavior from the care recipient (OR = 8.15), a difficult previous relationship (OR = 7.49), caregiver’s perception of burden (OR = 2.33), caregiver’s anxiety (OR = 2.01), caregiver’s depression (OR = 0.64), and functional dependency (OR = 0.58). The significant variables related to neglect were: caregiver’s perception of burden (OR = 2.63), caregiver’s anxiety (OR = 1.81), aggressive behavior from the care recipient (OR = 1.62), caregiver’s depression (OR = 1.51), positive aspects of caregiving (OR = 0.51), and age of the care recipient (OR = 0.52) (Table 5).

**Table 5:** Risk of mistreatment subtypes and associated factors. Logistic regression models: multivariate with all the variables and multivariate with significant variables

		Physical/psychological		Neglect	
		Multivariate LR^a^ Enter Method	Multivariate LR Final Model^d^	Multivariate LR Enter Method	Multivariate LR Final Model^e^
Variables		OR^b^ (IC95%^c^)	OR (IC95%)	OR (IC95%)	OR (IC95%)
Gender, caregiver	Male	1		1	
	Female	0.94 (0.55-1.62)		0.96 (0.60-1.56)	
Age, caregiver	20-44 y	1		1	
	45-64 y	0.72 (0.31-1.69)		1.50 (0.68-3.28)	
	65-74 y	0.91 (0.37-2.22)		0.96 (0.42-2.19)	
	75+ y.	0.56 (0.22-1.40)		1.71 (0.74-3.96)	
Gender, care-recipient	Male	1		1	
	Female	1.04 (0.67-1.61)		0.91 (0.61-1.34)	
Age, care-recipient	< 80 y.	1		1	1
	80-89 y	0.76 (0.46-1.26)		0.57 (0.36-0.89)	0.64 (0.43-0.95)
	90+ y.	0.54 (0.31-0.95)		0.55 (0.33-0.90)	0.52 (0.33-0.81)
Previous relationship	Good/Very good	1	1	1	
	Fair	2.17 (1.22-3.85)	2.19 (1.25-3.85)	0.99 (0.58-1.69)	
	Poor/Very poor	6.90 (1.38-34.5)	7.49 (1.57-35.8)	1.43 (0.45-4.57)	
Positive aspects of caregiving scale	1st quartile (min)	1		1	1
	2nd quartile	0.81 (0.48-1.36)		0.54 (0.34-0.88)	0.51 (0.32-0.81)
	3rd quartile	0.56 (0.32-0.98)		1.10 (0.66-1.85)	1.04 (0.63-1.73)
	4th quartile (max)	0.60 (0.35-1.04)		0.66 (0.41-1.08)	0.64 (0.40-1.03)
Has someone to count on	No	1		1	
	Yes	1.31 (0.84-2.05)		1.14 (0.76-1.71)	
Short Portable Mental Status Questionnaire	Intact cognition	1		1	
	Mild Impairment	1.65 (0.89-3.07)		0.98 (0.56-1.72)	
	Moderate Impairment	1.27 (0.71-2.25)		0.97 (0.58-1.62)	
	Severe Impairment	1.65 (0.94-2.88)		0.73 (0.45-1.20)	
Barthel Index	Moderate dependence	1	1	1	
	Severe dependence	0.74 (0.46-1.18)	0.75 (0.48-1.19)	0.71 (0.46-1.08)	
	Total dependence	0.49 (0.31-0.79)	0.58 (0.37-0.88)	0.98 (0.64-1.47)	
Goldberg depression	No	1	1	1	1
	Yes	0.61 (0.39-0.96)	0.64 (0.41-0.99)	1.50 (1.02-2.20)	1.51 (1.04-2.19)
Goldberg Anxiety	No	1	1	1	1
	Yes	1.92 (1.23-3.02)	2.01 (1.31-3.10)	1.91 (1.30-2.80)	1.81 (1.25-2.63)
Zarit Burden Scale	No burden	1	1	1	1
	Burden	2.27 (1.38-3.71)	2.33 (1.46-3.72)	2.70 (1.82-4.03)	2.63 (1.79-3.87)
Caregivers’ perception of aggressive behavior in the care recipient	No	1	1	1	1
	Yes	8.39 (5.62-12.5)	8.15 (5.54-11.9)	1.55 (1.10-2.19)	1.62 (1.16-2.26)

^a^*LR* Logistic regression, ^b^*OR* Odds Ratio, ^c^*IC95%* 95% confidence interval. ^d^Forward conditional approach Hosmer & Lemeshow test sig:0.95; *N* = 788. ^e^Forward conditional approach Hosmer & Lemeshow test sig:0.26; N = 788

***Positive aspects of caregiving scale*** of 9 items (range 5 to 45). Higher scores indicate greater perceived PAC

***Short Portable Mental Status Questionnaire*** *of* 10 items (range 0 from 10). Higher scores indicate worse mental status

***Barthel Index****.* Values range from 0 (completely dependent) to 100 (completely Independent)

***Goldberg depression*** of 9 items (range from 0 to 9). Values ≥2 have 50% chance of having clinically important disturbance. ***Goldberg Anxiety*** of 9 items (range from 0 to 9). Values ≥5 have 50% chance of having clinically important disturbance

***Zarit Burden Scale*** of 9 items. Values range from 5 to 45. Higher scores indicate more burden

## Discussion

The study examines the risk of abuse or mistreatment in the relationship between family caregivers and care recipients. The cohort of Spanish caregivers demonstrates that, in this setting, the role of caring is mainly performed by women with a low level of education and without a source of regular income, who live together with the care recipient. Such women are caregivers for many hours each day and over long periods of time. Paid caregivers were not included in the study because they are considered a different population with specific risk factors more related to training and workplace conditions. Our results regarding mistreatment need to be interpreted therefore within the context of a specific study population, that is to say, family caregivers of highly dependent elders, and taking into account the screening tool employed, the Caregiver Abuse Screen, that measures risk by means of an interview with the caregiver.

### Prevalence of mistreatment risk

We found that a third of the family caregivers acknowledged a high risk of engaging in mistreatment with respect to their care recipients. They had been acting as caregivers over considerable periods of time, and the risk of mistreatment due to burden, anxiety, or relationship problems was elevated. Data published on the prevalence of elder abuse by caregivers vary greatly [10]. Nonetheless, our study reported a very high percentage (33.4%) compared to other community-based studies, for example from the United States which describe a prevalence ranging from 5 to 10% [14–16]. Such variations could be partially explained by the different approaches used, in our case we estimated the risk of mistreatment rather than the mistreatment itself, or diverse study populations and varying screening tools. Our results are similar to those of Cooper et al. [18] and the WHO European Report. The latter indicates that the prevalence of elder abuse may be as high as 25% for older people with high dependency, and about one third of family caregivers report being involved in maltreatment [17].

### The measurement tool

Evaluating the risk of existing mistreatment by means of an interview is complex since it implies the measurement of interpersonal conflicts, multidimensional situations that require contextualization, and a reality that the actors attempt to conceal. A number of approaches, including direct questions and indicators obtained through observation, such as hygiene, malnutrition or bruises, aim to measure the existence of abuse. Another frequent line of action is the use of screening tests which are usually very sensitive but less specific.

Within the context of a population of dependent patients with a high percentage of dementia it was considered inadequate, or even difficult, to interview the care recipient. Consequently, in our study the CASE test for caregivers, an easily implementable screening tool already validated in the English and Spanish versions, was employed [34]. Even though it only indicates risk, the cut-off point of 4 has been consistently associated with a high possibility of mistreatment.

### Risk factors for elder mistreatment

Risk factors are associated with the characteristics of the care recipient, the caregiver, previous family relationships, and environmental factors [2, 12, 41].

### Characteristics of the care recipient

Functional dependency and cognitive impairment have been identified as risk factors consistently associated with care recipient abuse [2, 12, 16]. We have previously stated that our sample consisted of dependent care recipients, 64% because of dementia and 36% due to other causes. Interestingly, in our study greater cognitive impairment and functional dependency were not associated with a higher risk of mistreatment. On the contrary, we observed a higher risk of abusive behavior with moderate dependency, especially when the physical/psychological abuse subtype was analyzed. We need to emphasize yet again that, due to the characteristics of our sample, we cannot extrapolate our estimates to the independent or mildly dependent elders in the community. In that context, we suggest the possible existence of an inverted U shaped curve regarding risk of mistreatment and the level of functional dependency, where independent elders might be at lower risk of mistreatment than dependent ones. However, once dependency is total, risk of abuse lowers in comparison to that of less dependent elders. Individuals with less functional and cognitive impairment might present more disruptive behavior and greater interaction with the caregiver, which could be associated with a higher risk of abuse. Thus we found that when the caregiver reported aggressive behavior inflicted by the care recipient the risk of all types of abuse increased, nevertheless, such aggressive situations occurred less frequently when the dependency was total. A finding that corroborates the results of the review by Johannsen et al. [12] and other authors [18, 42] reporting that behavioral problems of the care recipients are a risk factor for abusive conduct in the caregiver. Our study does not show differences in the global risk of mistreatment based on the recipient’s gender or age. In contrast, in a North American study, Laumann et al. observed that women and the less elderly reported suffering verbal abuse more frequently [15].

### Caregiver burden and mood disorders

In our study, the proportion of caregivers with feelings of burden and scores in Goldberg’s scale suggestive of anxiety and depression was over 40%, confirming the negative impact of the family caregiver role. It is clear that such sensations have been associated with a higher risk of mistreatment, indeed, they may translate into experiencing tension, which could result in inappropriate behavior when caring for the elderly. Depression symptoms could also hinder appropriate care and lead to situations of neglect. Such data are in agreement with the results obtained in the recent review by Boye et al. [43] and the studies of Cooper et al. [28, 44], Johannesen [12], and Pérez-Rojo et al. [45]. In the subtypes of mistreatment analyses, caregiver depression was more associated with the presence of neglect risk, possibly explained by the characteristics of depression symptoms, such as fatigue and inhibition.

### Social support

We highlight social support as an essential factor, since the caregivers who explained that they did not have any help were at a higher risk of perpetrating abusive behavior. In fact, in accordance with the classic risk factors of mistreatment, social isolation is an ideal breeding ground for the development of abusive situations [46].

### Protective factors: Previous relationship and perception of positive aspects of care

We also found protective factors, namely: loving, respectful relationships prior to the dependency, social support, and a greater awareness of the positive aspects of care. In agreement with other studies with regard to the relationship between caregivers and care recipients, we found that caregivers with a good prior relationship perceived the ongoing interaction and their role as less stressful, which in turn decreased the risk of abusive behavior [22, 31, 47]. In addition, several authors point out that both institutional and informal support reduces the risk of mistreatment [14, 21, 48].

### Limitations of the study

Our study presents some limitations. They are mainly due to the fact that it includes family caregivers of elderly people who present moderate to severe dependency, with a high prevalence of cognitive impairment. In consequence, the results are only applicable to caregivers of similar characteristics. On the other hand, the frequency of this profile and the evidence that they are a population at risk of engaging in abusive behavior justifies their inclusion in this study.

We have previously mentioned that CASE is a measurement of risk of abuse, but cannot verify whether mistreatment does actually occur. Furthermore, CASE is a tool with high sensitivity but low specificity, and does not screen for sexual abuse or financial exploitation. Nevertheless, we considered it the best available instrument to approach our object of study because it has been validated in our setting and due to the characteristics of the care recipients. In addition, the CASE was administered in a friendly environment and the interviewers contextualized the questions, thus increasing their specificity.

### Implications of results. Possible interventions

Risk of mistreatment is high among dependent elderly individuals. We emphasize the need to raise awareness of this issue amongst health and social care professionals, increasing the screening activities for early detection and secondary intervention. Detection of elder abuse should be followed up by an intervention plan and close monitoring. A recent systematic review of interventions to prevent and cease elder abuse only showed effectiveness when targeting physical restraint by long-term paid caregivers; there is still a lack of evidence for interventions focusing on abusive family caregivers [49].

Nevertheless, our study has found risk factors in family caregivers that to some extent are preventable, namely anxiety and feelings of burden. Primary prevention activities to reduce risk factors should be individually-focused or community-based interventions. Psychosocial interventions, such as support groups, may have some efficacy in lowering caregiver burden as reported in a review by Adelman [50]. Moreover, there is some evidence from a recent Cochrane review [51] that interventions to promote the mental health of family caregivers may improve their anxiety and depression levels. Whether such programs also reduce occurrence or recurrence of abuse needs to be further studied. It is, however, essential to become aware of these risk factors and their causes (for instance, lack of family or social/health service support) in order to intervene. Interventions that can have a significant impact on the wellbeing of both caregivers and care recipients include education and training, financial assistance for dependency cases, adequate social support, and respite periods for the caregiver. Such actions would create a breathing space for the families to protect their elders and decrease the risk of mistreatment.

## Conclusions

Prevalence of risk of mistreatment is high among family caregivers. Our study has found risk factors in family caregivers that are preventable to an extent, namely: anxiety and feelings of burden. It is essential to become aware of these risk factors and their causes to intervene and help primary as well secondary prevention.

## Appendix 1

### Caregivers’ perception of aggressive behavior in the care recipient questionnaire

Five questions (with response yes/no) designed ad hoc to evaluate the caregivers’ perception of aggressive or disruptive behavior in the care recipient.

1. Does he/she ever control you?
2. Does he/she ever scold you?
3. Does he/she ever yell at you?
4. Does he/she ever threaten you?
5. Has he/she ever hurt you?

## Abbreviations

CASE: Caregiver abuse screen
CI: Confidence interval
NCEA: National center on elder abuse
OR: Odds ratio
PAC: Positive aspects of caregiving
SPMSQ: Short portable mental status questionnaire

## References

[1] Lachs MS, Pillemer K (2004). Elder abuse. Lancet.

[2] Lachs MS, Pillemer KA (2015). Elder Abuse. N Engl J Med.

[3] World Health Organization. Elder Abuse Fact sheets. 2016. Available from: http://www.who.int/mediacentre/factsheets/fs357/en/

[4] CDC. Understanding Elder Abuse: 2015 Fact Sheet. 2016. Available from: https://www.cdc.gov/violenceprevention/pdf/em-factsheet-a.pdf

[5] Pillemer K, Burnes D, Riffin C, Lachs MS (2016). Elder abuse: global situation, risk factors, and prevention strategies. Gerontologist.

[6] Dong X, Simon M, Beck T, Farran C, McCann J, Mendes de Leon C (2011). Elder abuse and mortality: the role of psychological and social wellbeing. Gerontology.

[7] Lachs MS, Williams CS, O’Brien S, Pillemer KA, Charlson ME (1998). The mortality of elder mistreatment. JAMA.

[8] Mosqueda L, Dong X (2011). Elder abuse and self-neglect: “I don’t care anything about going to the doctor, to be honest...”. JAMA.

[9] Schofield MJ, Powers JR, Loxton D (2013). Mortality and disability outcomes of self-reported elder abuse: a 12-year prospective investigation. J Am Geriatr Soc.

[10] Dong X (2015). Elder abuse: systematic review and implications for practice. J Am Geriatr Soc.

[11] Cooper C, Selwood A, Livingston G (2009). Knowledge, detection, and reporting of abuse by health and social care professionals: a systematic review. Am J Geriatr Psychiatry.

[12] Johannesen M, LoGiudice D (2013). Elder abuse: a systematic review of risk factors in community-dwelling elders. Age Ageing.

[13] Vetere PM (2011). Elder abuse: what are we missing?. Can Fam Physician.

[14] Acierno R, Hernandez MA, Amstadter AB, Resnick HS, Steve K, Muzzy W (2010). Prevalence and correlates of emotional, physical, sexual, and financial abuse and potential neglect in the United States: the National Elder Mistreatment Study. Am J Public Health.

[15] Laumann EO, Leitsch SA, Waite LJ (2008). Elder mistreatment in the United States: prevalence estimates from a nationally representative study. J Gerontol B Psychol Sci Soc Sci.

[16] Burnes D, Pillemer K, Caccamise PL, Mason A, Henderson CR, Berman J (2015). Prevalence of and risk factors for elder abuse and neglect in the community: a population-based study. J Am Geriatr Soc.

[17] World Health Organization. European report on preventing elder maltreatment. Copenhagen; 2011. Available from: http://www.euro.who.int/__data/assets/pdf_file/0010/144676/e95110.pdf

[18] Cooper C, Selwood A, Livingston G (2008). The prevalence of elder abuse and neglect: a systematic review. Age Ageing.

[19] Wiglesworth A, Mosqueda L, Mulnard R, Liao S, Gibbs L, Fitzgerald W (2010). Screening for abuse and neglect of people with dementia. J Am Geriatr Soc.

[20] Cooper C, Selwood A, Blanchard M, Walker Z, Blizard R, Livingston G (2009). Abuse of people with dementia by family carers: representative cross sectional survey. BMJ.

[21] Garre-Olmo J, Planas-Pujol X, López-Pousa S, Juvinya D, Vilà A, Vilalta-Franch J (2009). Prevalence and risk factors of suspected elder abuse subtypes in people aged 75 and older. J Am Geriatr Soc.

[22] Pérez-Cárceles M, Rubio L, Pereniguez JE, Pérez-Flores D, Osuna E, Luna A (2009). Suspicion of elder abuse in South Eastern Spain: the extent and risk factors. Arch Gerontol Geriatr.

[23] Pérez-Rojo G, Izal M, Montorio I, Regato P, Espinosa J (2013). Prevalence of elder abuse in Spanish dwelling in community. Med Clin (Barc).

[24] Risco Romero C, del Carmen Paniagua Vicioso M, Jiménez Mendoza G, Poblador Curtó MD, Molina Martínez L, Buitrago F (2005). Prevalence and risk factors of suspicion abuse in elder population. Med Clin (Barc).

[25] Ruiz Sanmartín A, Altet Torner J, Porta Martí N, Duaso Izquierdo P, Coma Solé M, Requesens TN (2001). Domestic violence: prevalence of suspected ill treatment of the elderly. Aten Primaria.

[26] Iborra I. Elder Abuse in the Family in Spain. Valencia: Fundación de la Comunitat Valenciana para el estudio de la violencia (Centro Reina Sofía); 2008. Available from: http://www.inpea.net/images/Espana_Informe_2008_Maltrato.pdf

[27] Pérez-Rojo G, Izal M, Montorio I, Nuevo R (2008). Identificación de factores de riesgo de maltrato hacia personas mayores en el ámbito comunitario. Int J Clin Heal Psychol.

[28] Cooper C, Blanchard M, Selwood A, Walker Z, Livingston G (2010). Family carers’ distress and abusive behaviour: longitudinal study. Br J Psychiatry.

[29] Lachs MS, Williams C, O’brien S, Hurst L, Horwitz R (1997). Risk factors for reported elder abuse and neglect: a nine-year observational cohort study. Gerontologist.

[30] Cohen M, Halevi-Levin S, Gagin R, Friedman G (2006). Development of a screening tool for identifying elderly people at risk of abuse by their caregivers. J Aging Health.

[31] Reis M, Nahmiash D (1998). Validation of the indicators of abuse (IOA) screen. Gerontologist.

[32] Fulmer T, Guadagno L, Bitondo Dyer C, Connolly MT (2004). Progress in elder abuse screening and assessment instruments. J Am Geriatr Soc.

[33] Reis M, Nahmiash D (1995). Validation of the caregiver abuse screen (CASE). Can J Aging.

[34] Pérez-Rojo G, Nuevo R, Sancho M, Penhale B (2015). Validity and reliability of the Spanish version of caregiver abuse screen (CASE). Res Aging.

[35] Baztán J, Pérez del Molino J, Alarcón T, San Cristóbal E, Izquierdo G, Manzarbeitia J (1993). Índice de Barthel: Instrumento válido para la valoración funcional de pacientes con enfermedad cerebrovascular. Rev Esp Geriatr Gerontol.

[36] Martínez de la Iglesia J, Dueñas Herrero R, Onís Vilches MC, Aguado Taberné C, Albert Colomer C, Luque Luque R (2001). Spanish language adaptation and validation of the Pfeiffer’s questionnaire (SPMSQ) to detect cognitive deterioration in people over 65 years of age. Med Clin (Barc).

[37] Regueiro Martínez AA, Pérez-Vázquez A, Gómara Villabona SM, Ferreiro Cruz MC (2007). Short Zarit interview on burden of care for caregivers in primary care. Aten Primaria.

[38] Montón C, Pérez Echeverría MJ, Campos R, García Campayo J, Lobo A (1993). Anxiety scales and Goldberg’s depression: an efficient interview guide for the detection of psychologic distress. Aten Primaria.

[39] Tarlow BJ, Wisniewski SR, Belle SH, Rubert M, Ory MG, Gallagher-Thompson D (2004). Positive aspects of Caregiving: contributions of the REACH project to the development of new measures for Alzheimer’s Caregiving. Res Aging.

[40] Hosmer DW, Lemeshow S, Sturdivant RX, JOHN WILEY & SONS,INC. (2013). Applied logistic regression.

[41] Wang XM, Brisbin S, Loo T, Straus S (2015). Elder abuse: an approach to identification, assessment and intervention. Can Med Assoc J.

[42] Lee M, Kolomer S (2005). Caregiver Burden, Dementia, and Elder Abuse in South Korea. J Elder Abuse Negl.

[43] Boye F, Yan E. Abuse of Older Persons With Dementia. Trauma Violence Abus. 2016. Available from: http://journals.sagepub.com/doi/10.1177/1524838016650185 10.1177/1524838016650185 27247138

[44] Cooper C, Selwood A, Blanchard M, Walker Z, Blizard R, Livingston G (2010). The determinants of family carers’ abusive behaviour to people with dementia: results of the CARD study. J Affect Disord.

[45] Pérez-Rojo G, Izal M, Montorio I, Penhale B (2009). Risk factors of elder abuse in a community dwelling Spanish sample. Arch Gerontol Geriatr.

[46] Dong X, Simon M, Gorbien M, Percak J, Golden R (2007). Loneliness in older Chinese adults: a risk factor for elder mistreatment. J Am Geriatr Soc.

[47] Cooney C, Howard R, Lawlor B (2006). Abuse of vulnerable people with dementia by their carers: can we identify those most at risk?. Int J Geriatr Psychiatry.

[48] Dong X, Simon M (2008). Is greater social support a protective factor against elder mistreatment?. Gerontology.

[49] Ayalon L, Lev S, Green O, Nevo U (2016). A systematic review and meta-analysis of interventions designed to prevent or stop elder maltreatment. Age Ageing.

[50] Adelman RD, Tmanova LL, Delgado D, Dion S, Lachs MS (2014). Caregiver Burden. JAMA.

[51] Baker PR, Francis DP, Hairi NN, Othman S, Choo WY (2016). Interventions for preventing abuse in the elderly. Cochrane Database Syst Rev.

